# Seroprevalence and risk factors of COVID-19 in healthcare workers from 11 African countries: a scoping review and appraisal of existing evidence

**DOI:** 10.1093/heapol/czab133

**Published:** 2021-11-02

**Authors:** Sophie Alice Müller, Rebekah Ruth Wood, Johanna Hanefeld, Charbel El-Bcheraoui

**Affiliations:** Centre for International Health Protection, Robert Koch Institute, Nordufer 20, Berlin 13353, Germany; Evidence-Based Public Health, Centre for International Health Protection, Robert Koch Institute, Nordufer 20, Berlin 13353, Germany; Centre for International Health Protection, Robert Koch Institute, Nordufer 20, Berlin 13353, Germany; Evidence-Based Public Health, Centre for International Health Protection, Robert Koch Institute, Nordufer 20, Berlin 13353, Germany

**Keywords:** Scoping review, seroprevalence, Africa, COVID-19, SARS-CoV-2, health professionals

## Abstract

A better understanding of serological data and risk factors for coronavirus disease 2019 (COVID-19) infection in healthcare workers (HCWs) is especially important in African countries where human resources and health services are more constrained. We reviewed and appraised the evidence of severe acute respiratory syndrome coronavirus 2 (SARS-CoV-2) seroprevalence and its risk factors in HCWs in Africa to inform response and preparedness strategies during the SARS-CoV-2 pandemic. We followed the Preferred Reporting Items for systematic reviews and Meta-Analyses extension for Scoping Reviews (PRISMA-ScR) guidelines in this scoping review. Databases including PubMed, Embase and preprint servers were searched accordingly from the start of the COVID-19 pandemic to 19 April 2021. Our search yielded 12 peer-reviewed and four pre-print articles comprising data on 9223 HCWs from 11 countries in Africa. Seroprevalence varied widely and ranged from 0% to 45.1%. Seropositivity was associated with older age, lower education, working as a nurse/non-clinical HCW or in gynaecology, emergency, outpatient or surgery departments. Asymptomatic rates were high and half of the studies recommended routine testing of HCWs. This scoping review found a varying but often high SARS-CoV-2 seroprevalence in HCWs in 11 African countries and identified certain risk factors. COVID-19 public health strategies for policy and planning should consider these risk factors and the potential for high seroprevalence among HCWs when prioritizing infection prevention and control measures and vaccine deployment.

**Key messages**

- There are 16 articles on seroprevalence comprising data on 9223 HCWs from 11 countries in Africa.
- Seroprevalence varied widely and ranged from 0% to 45.1%.
- Seropositivity was associated with older age, lower education, working as a nurse/non-clinical HCW, or in gynaecology, emergency, outpatient, or surgery departments.
- COVID-19 policies should consider these risk aspects and the partly high seroprevalence among HCWs when prioritizing IPC measures and vaccine deployment.

## Introduction

The coronavirus disease 2019 (COVID-19) pandemic has put health systems worldwide to an unprecedented test. As frontline workers, healthcare workers (HCWs) are a critical part of the health system’s pandemic response and are themselves at high risk of infection. Vaccine roll-out, therefor, prioritizes HCWs worldwide (WHO, 2020b), and at the time of writing, 35 African countries had begun vaccine campaigns (Our World in Data, 2021).

Serological studies are investigations using serology testing to look for antibodies in blood (Centers for Disease Control and Prevention, 2021). These studies give insight into the true burden of infection as they have the ability to account for asymptomatic and unreported cases. These studies can provide data on infection trends, effects of interventions, vaccine deployment prioritization guidelines (Maeda and Nkengasong, 2021) and geographical distribution mapping for identification of populations at particularly high risk (Peeling *et al.*, 2020).

A previous systematic review on seroprevalence in HCWs only included three African studies (Galanis *et al.*, 2020). Nevertheless, data on seroprevalence and risk factors are specifically important in the African continent, where countries have predominantly lower access or quality of healthcare (Our-World-in-Data, 2015). This region furthermore experiences constrained human resources in healthcare. Compared to Europe, where there are 80 nurses per 10 000 population, across Africa there are only 10 (WHO, 2021a), emphasizing the high need to protect this scare workforce. Evidence on the implementation of measures that can help detect and mitigate exposures such as triage systems, dedicated COVID-19 wards, as well as surveillance testing and infection prevention and control (IPC) availability is necessary to better understand the risk of becoming overburdened.

This need is further emphasized in a region that does not have ownership of vaccine production and must depend on limited doses from other areas. Serological testing can also provide guidance on vaccine decision-making.

This scoping review aimed to better understand and identify gaps in knowledge on seroprevalence of severe acute respiratory syndrome coronavirus 2 (SARS-CoV-2) antibodies in HCWs in the African continent and their risk factors for COVID-19 infection, as well as how these seroprevalence rates compare with the respective general population. The added public health value of this review is that it provides the most up to-date synthesis of all available research and hence can inform public health strategies for policy and planning.

## Materials and methods

Given the relative infancy and fast-moving pace of the field, we selected a scoping review method without prior registration in a public domain.

### Data sources and search strategy

This scoping review was performed according to the Preferred Reporting Items for systematic reviews and Meta-Analyses extension for Scoping Reviews (PRISMA-ScR) Checklist (Tricco *et al.*, 2018) and frameworks on scoping reviews (Levac *et al.*, 2010; Arksey and O’malley, 2005; Peters Mdj *et al.*, 2020) including the stages of (1) identifying a research question, (2) identifying relevant studies, (3) study selection, (4) charting the data and (5) summarizing and reporting results. The detailed PRISMA checklist is available in Supplementary Table S1.

We searched PubMed, Embase and preprint servers (ArRvix, BioRvix, ChemRvix, MedRvix, Preprints.org, ResearchSquare and SSRN) for designated terms for COVID-19, seroprevalence, HCWs and African countries. The complete search strategy is detailed in supplement 2. A hand-search was also conducted to add relevant articles not previously identified with the search strategy. Additionally, to put the HCW seroprevalences into context, general population seroprevalences were gathered from the SeroTracker database (Arora *et al.*, 2021) for all countries included on 24 October 2021.

### Selection and eligibility criteria

We screened title and abstracts of all publications returned in the search, guided by pre-set inclusion and exclusion criteria detailed as follows. We included articles published or in preprint between the first detection of COVID-19 and 19 April 2021 on HCWs irrespective of prior COVID-19 status or comorbidities in health settings in Africa with data on serological status. We excluded reviews and evidence that focused on non-HCWs or outside of Africa. HCWs were not defined in this scoping review, but we followed the definition of included studies, being mainly ‘clinical and non-clinical care’. We did not restrict our search in terms of publication language, although search terms were all in English. Two researchers independently undertook study selection, and discussion resolved potential disagreement. Selection of studies for general population seroprevalence was restricted to the respective counties and overall study period.

### Data extraction and quality assessment

Data from all selected studies were entered independently by two researchers into a form created for this purpose in Microsoft Excel. The following data were charted: author, publication year, title, journal, publication status, study period, city/country, sample size, sampling strategy, response rate, inclusion criteria, exclusion criteria, study design, sex, age, seroprevalence, asymptomatic rate, sample collection, testing strategy, testing rate, type of antibody, type of test, test name, factors investigated, factors associated, level of analysis and additional data such as reported triage system, dedicated COVID-19 wards, routine testing, IPC or personal protective equipment (PPE) availability.

The quality of studies was assessed by two authors independently using Joanna Briggs Institute (JBI) critical appraisal tool for prevalence studies (Munn *et al.*, 2015) adapted with additional specifications (Arora *et al.*, 2021). Bias levels were assessed using a 9-point ranking system with categorization of 8–9 low, 5–7 moderate and ≤4 high risk of bias (Galanis *et al.*, 2020).

## Results

### Selection of studies

**Figure 1.**
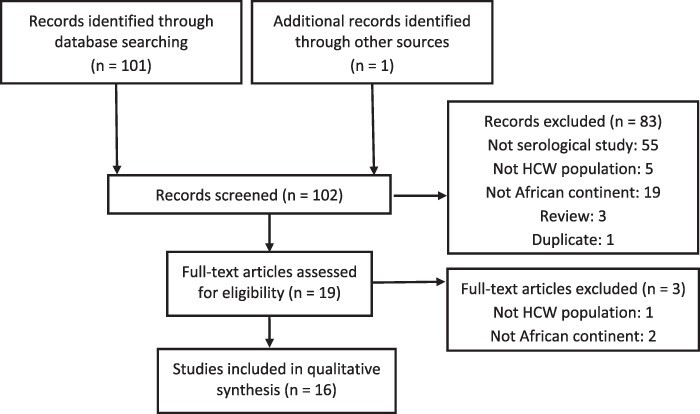
Selection of sources of evidence

The search identified a total of 102 articles; following the removal of duplicates and critical assessment of title and abstracts, 19 potentially relevant articles were identified for full-text evaluation (Figure 1). Application of the pre-set eligibility criteria resulted in a final inclusion of 16 articles.

### Study characteristics

The included 16 studies contain seroprevalence data on 9223 HCWs from 11 countries across Africa: Democratic Republic of Congo (Mukwege *et al.*, 2021), Egypt (Mostafa *et al.*, 2020; 2021; Abdelmoniem *et al.*, 2021; Kassem *et al.*, 2020; Mukhtar *et al.*, 2021), Kenya (Etyang *et al.*, 2021), Libya (Kammon *et al.*, 2020), Malawi (Chibwana *et al.*, 2020), Mauretania (Salem *et al.*, 2021), Nigeria (Majiya *et al.*, 2020; Olayanju *et al.*, 2020), South Africa (Goldblatt *et al.*, 2021), Togo (Halatoko *et al.*, 2020), Zambia (Fwoloshi *et al.*, 2021) and Zimbabwe (Rusakaniko *et al.*, 2021) (Table 1).

**Table 1.** Results of included studies

Reference	City, country	Time	Sample size (*n*)	Seroprevalence % (95% CI)	Asymptomatic rate %	Factors investigated	Highest level of analysis
Abdelmoniem *et al.* (2021)	Cairo, Egypt	06/2020	203	18.2 (13.2–24.2)	NA^a^	Demographics (age, gender), profession (occupation), exposure (contact with case), IPC questionnaires (use of PPE, HH), medical condition (comorbidities)	Univariate
Chibwana *et al.* (2020)	Blantyre, Malawi	05/2020–06/2020	500	16.8 (13.6–20.4)	NA^a^	None	NA^a^
Etyang *et al.* (2021)	Kilifi, Busia; Nairobi, Kenya	07/2020–12/2020	684	20.8 (17.5–24.4)	NR^b^	Demographics (age, gender), profession (occupation), exposure (**site**, working in COVID unit), medical condition (chronic illness), symptoms	Multivariate
Fwoloshi *et al.* (2021)	6 districts, Zambia	07/2020	575	2.2 (0.5–3.9)	NR^b^	Demographics (age, gender), **profession** (occupation), exposure (**district**, health facility type, contact, direct patient care, care for confirmed COVID-19, travel, visit of health facility, **in-person attendance at work/school**, **market visit**, transportation), medical condition (**unknown HIV status**, pregnancy)	Univariate
Goldblatt *et al.* (2021)	Cape Town, South Africa	06/2020–08/2020	222	10.4 (6.7–15.1)	68.9	None	NA^a^
Halatoko *et al.* (2020)	Lomé, Togo	04/2020–05/2020	370	1.4 (0.4–3.1)	NR^b^	None	NA^a^
Kammon *et al.* (2020)	Alzintan, Libya	04/2020–05/2020	77	0 (0.0–4.7)	NR^b^	None	NA^a^
Kassem *et al.* (2020)	Cairo, Egypt	06/2020	74	12.2 (5.7–21.8)	62.5	Demographics (age, gender), profession (occupation), exposure (contact with case/suspect), IPC questionnaires (use of PPE, HH), medical condition (comorbidities), symptoms	Univariate
Majiya *et al.* (2020)	Niger State, Nigeria	06/2020	43	37.2 (23.0–53.3)	NR^b^	None	NA^a^
Mostafa *et al.* (2020)	Cairo, Egypt	04/2020–05/2020	4040	1.3 (1.0–1.7)	68.2	Demographics (**age**, gender, residence, marital status, education), profession (**occupation, department**), medical condition (immunological disorder, tobacco use), exposure (location of contact with confirmed/suspected case, duration, type of contact), symptoms (severity, **fever, cough, change**/**loss of smell**/taste/appetite)	Multivariate
Mostafa *et al.* (2021)	Cairo, Egypt	05/2020–06/2020	2282	4.0 (3.6–5.3)	64.0	Demographics (**age**, gender, residence, marital status, **education**), profession (occupation), exposure (**location of contact with confirmed/suspected case**, duration, type of contact), medical condition (**comorbidities, pregnancy**, tobacco use), symptoms (severity, fever, muscle pain, joint pain, sneezing, shortness of breathing, other respiratory symptoms, loss of appetite, change/loss of taste, **change/loss of smell**, conjunctivitis)	Multivariate
Mukhtar *et al.* (2021)	Cairo, Egypt	05/2020–06/2020	455	7.9 (5.8–10.8)	31.0	Demographics (age, gender), medical condition (clinical history, medication intake, smoking history), symptoms	Univariate
Mukwege *et al.* (2021)	Bukavu, DRC	07/2020–08/2020	359	41.2 (36.1–46.5)	77.7	Demographics (gender), profession (occupation), exposure (type of work, use of PPE, contact with confirmed case), medical condition (comorbidities), **confirmed case**	Multivariate
Olayanju *et al.* (2020)	Ibadan, Nigeria	04/2020	133	45.1 (36.5–54.0)	NA^a^	Demographics (age, gender), profession (occupation, **department**)	Univariate
Rusakaniko *et al.* (2021)	Bulawayo, Zimbabwe	06/2020	635	26.1 (22.8–29.7)	NR^b^	Demographics (**age**), profession (**occupation, department**), medical condition (**comorbidities**)	Univariate
Salem *et al.* (2021)	Nouakchott, Mauritania	05/2020	853	1.7 (0.9–2.7)	NR^b^	None	NA^a^

a NA: not applicable as the study excluded symptomatic HCWs.

b NR: not reported, bolded factors are those significantly associated with seropositivity.

Twelve articles were published in peer-reviewed journals (Goldblatt *et al.*, 2021; Halatoko *et al.*, 2020; Abdelmoniem *et al.*, 2021; Kassem *et al.*, 2020; Mostafa *et al.*, 2020; Olayanju *et al.*, 2020; Mostafa *et al.*, 2021; Mukwege *et al.*, 2021; Mukhtar *et al.*, 2021; Salem *et al.*, 2021; Fwoloshi *et al.*, 2021; Rusakaniko *et al.*, 2021) and four were published in preprint services (Chibwana *et al.*, 2020; Kammon *et al.*, 2020; Majiya *et al.*, 2020; Etyang *et al.*, 2021). Twelve studies used total population sampling, reflecting that all HCWs of a given setting were included (Chibwana *et al.*, 2020; Goldblatt *et al.*, 2021; Abdelmoniem *et al.*, 2021; Kassem *et al.*, 2020; Mostafa *et al.*, 2020; 2021; Etyang *et al.*, 2021; Mukhtar *et al.*, 2021; Salem *et al.*, 2021; Mukwege *et al.*, 2021; Fwoloshi *et al.*, 2021; Rusakaniko *et al.*, 2021) and four reported to have used a random sampling strategy (Halatoko *et al.*, 2020; Kammon *et al.*, 2020; Majiya *et al.*, 2020; Olayanju *et al.*, 2020). All studies were conducted between April and December of 2020 in hospital settings, with the exception of two studies which did not report details on HCW recruitment (Halatoko *et al.*, 2020; Majiya *et al.*, 2020). Three studies took place in specific departments, such as emergency (Abdelmoniem *et al.*, 2021), paediatrics (Goldblatt *et al.*, 2021) or gastroenterology (Kassem *et al.*, 2020). In six studies, the percentage of males was higher than the percentage of females (Kammon *et al.*, 2020; Abdelmoniem *et al.*, 2021; Majiya *et al.*, 2020; Mukhtar *et al.*, 2021; Mukwege *et al.*, 2021; Salem *et al.*, 2021).

Reported sensitivity ranged from 71.1% (Mukwege *et al.*, 2021) to 100% (Goldblatt *et al.*, 2021) and specificity from 85.0% (Halatoko *et al.*, 2020) to 100% (Fwoloshi *et al.*, 2021; Mostafa *et al.*, 2020; 2021; Mukwege *et al.*, 2021). More than 60% of the studies used tests that met the commonly set minimum performance criteria of sensitivity ≥90% and test specificity >95% (Biocentury, 2020). Please see Supplementary Table S5 for detailed information on all study and antibody test characteristics discussed above.

Six studies assessed rates of asymptomatic infection, five of which reported asymptomatic rates above 60% (Goldblatt *et al.*, 2021; Kassem *et al.*, 2020; Mostafa *et al.*, 2021; 2020).

### Quality assessment

Following the JBI critical appraisal tool, studies were rated out of a total of 9 points, and all were found to be of moderate-to-low risk of bias (ranging between 5 and 8) (Supplementary Table S3). Risk of bias primarily arose from aspects such as test characteristics or non-reporting of response rates (Supplementary Table S3).

### Seroprevalence and its geographic distribution

Seroprevalence among studies ranged from 0% to 45.1%, with highest prevalence in Nigeria (45.1%) (Olayanju *et al.*, 2020) and DRC 41.2% (Mukwege *et al.*, 2021) and lowest in Libya, Togo and Egypt (0%, 1.4% and 1.3%, respectively) (Kammon *et al.*, 2020; Halatoko *et al.*, 2020; Mostafa *et al.*, 2020). Data collection across countries started in April 2020 and ended in December 2020 (Table 1). Five studies originated from Egypt, four of which were cross-sectional, while one was a consecutive follow-up cohort study for one of the three studies (Mostafa *et al.*, 2021). The follow-up cohort study found a seroconversion rate of 4% at a 3-week interval (Mostafa *et al.*, 2021), being 2.7 percentage points higher than the baseline cross-sectional study finding of 1.3% seroprevalence (Mostafa *et al.*, 2020). The three other cross-sectional studies found a prevalence of 12.2%, 18.2% and 31.0% among HCWs (Kassem *et al.*, 2020; Abdelmoniem *et al.*, 2021; Mukhtar *et al.*, 2021).

### Risk factors

Ten studies investigated factors associated with seropositivity, eight of which found association reported by odds ratio (OR) or hazard ratio (HR) (Table 1).

Risk of seropositivity increased with age. This was shown in detail in two studies from Egypt with the exemption of age group 40–49 years [Mostafa *et al.*, 2020: reference age: 18–24 years; 25–29 years, OR = 3.9, confidence interval (CI) 0.96–16.1; 30–39 years, OR = 4.3, CI 1.1–17.3; 40–49 years, OR 2.8, CI 0.6–12.9; ≥50 years, OR = 4.2, CI 0.8–21.2 and Mostafa *et al.*, 2021: reference age 18–29 years; 30–39 years, HR = 2.6, CI 1.3–4.9; 40–49 years, HR = 2.4, CI 1.2–4.7; ≥50 years, HR = 2.7, CI 1.3–5.6]. Sex was not associated with seropositivity. Lower levels of education had higher risks for seropositivity (reference education: university or higher; secondary HR = 2.0, CI 1.2–3.3; primary/preparatory HR = 3.9, CI 1.9–8.0; less than primary HR = 3.3, CI 1.4–7.8; Mostafa *et al.*, 2021). Odds of seropositivity were higher for HCWs in the operating room (OR = 3.2, CI 1.3–8.0; Mostafa *et al.*, 2020), emergency department (OR = 3.2, CI 1.1–9.4; Olayanju *et al.*, 2020), outpatient department (OR = 2.3, CI not reported; Rusakaniko *et al.*, 2021) or obstetrics and gynaecology department (OR = 19.3, CI 2.0–183.4; Olayanju *et al.*, 2020). Two studies reported higher risk of seropositivity for nurses (OR = 4.7, CI 2.0–11.2; OR = 3.0, CI 1.6–5.5; reference group physicians, respectively; Mostafa *et al.*, 2020; 2021), and one study reported higher risks for non-clinical HCWs (OR 7.8, CI 1.7–34.9, reference group nurses; Fwoloshi *et al.*, 2021).

Symptoms like fever, dry cough and change/loss of smell were associated with seropositivity (Kassem *et al.*, 2020; Mostafa *et al.*, 2020; 2021), with change/loss of smell having the highest odds (OR = 26.2, CI 2.1–329.7; Mostafa *et al.*, 2020). Medical conditions like pregnancy and chronic kidney disease were associated with seropositivity (OR = 3.5, CI 1.1–11.9; OR = 4.4, CI 1.0–19.0, respectively; Mostafa *et al.*, 2021). More than 15 minutes of contact with a confirmed case were positively associated (OR = 2.2, CI 1.2–4.1) with seropositivity, whereby exposure at work was negatively associated (OR = 0.5, CI 0.3–0.8; Mostafa *et al.*, 2021). No association between seropositivity and use of IPC measures could be found, but in-person attendance at work/school and frequent market visits (3–5 visits/month) were found to be risk factors (OR = 4.8, CI 1.6–13.9; OR = 3.0, CI 1.3–7.0, respectively; Fwoloshi *et al.*, 2021).

### Gap analysis

None of the studies reported facilities having a COVID-19 triage in place. Only one specified existence of dedicated wards (Kassem *et al.*, 2020) or that HCWs were specifically trained in COVID-19 IPC measures (Fwoloshi *et al.*, 2021). Five studies raised concerns about insufficient PPE availability or asked to increase supply (Majiya *et al.*, 2020; Mukwege *et al.*, 2021; Etyang *et al.*, 2021; Fwoloshi *et al.*, 2021; Rusakaniko *et al.*, 2021), whereby one study asked for PPE especially for HCWs with patient contact (Rusakaniko *et al.*, 2021). Fifty percent of included studies stated a lack of routine testing of HCWs or recommended its implementation due to the high seroprevalence and asymptomatic rates.

### General population seroprevalence comparison

Seroprevalence in the general population in the included countries ranged between 0% (Elfakhri *et al.*, 2020) and 44.6% (Marion *et al.*, 2021). For four countries, there were no general population data available: Malawi, Mauritania, Togo and Zimbabwe. Seroprevalence in the other included countries ranged from 16.6% (Nkuba *et al.*, 2021) to 40.8% (Philippe *et al.*, 2021) in DRC, from 29.8% (Girgis *et al.*, 2021) to 41.0% (Musa *et al.*, 2021) in Egypt, from 1.3% (Lucinde *et al.*, 2021) to 34.7% (Ngere *et al.*, 2021) in Kenya, from 0% (Elfakhri *et al.*, 2020) to 4.2% (Kammon *et al.*, 2020) in Libya, from 16.1% (Okpala *et al.*, 2021) to 42.0% (Ijeoma *et al.*, 2021) in Nigeria, from 23% (Kleynhans *et al.*, 2021) to 44.6% (Marion *et al.*, 2021) in South Africa and from 2.1% (Mulenga *et al.*, 2021) to 8.2% (Hines *et al.*, 2021) in Zambia.

## Discussion

The overall seroprevalence among HCWs across 11 Africa countries varied widely reflecting recent data from a systematic review of seroprevalence of anti-SARS-CoV-2 antibodies in Africa, which combined the general population and specific working groups (Chisale *et al.*, 2021).

Direct comparison with the general population is challenging and was not the primary aim of the current manuscript, but data available from the general population in the 11 included countries suggest highly varying seroprevalences similar to those of HCWs with rates between 0% and 44.6%. The seroprevalence among HCWs could be influenced by variety of factors that may differ between levels (national and local) and countries. National Public Health Institutes vary widely in breadth and depth (Africa CDC, 2019), especially in West Africa, where the strengthening of public health structures (Meda *et al.*, 2016) and surveillance is urgently needed (Massinga Loembé *et al.*, 2020). This need could be reflected in the suspected underreporting of SARS-CoV-2 cases. The Africa Centres for Disease Control and Prevention (CDC) together with Ministries of Health have recognized this challenge and taken action with their joint continental Strategy and the creation of Africa Task Force for Coronavirus (Africa CDC, 2020).

HCWs in Africa are potentially more often in contact with undiagnosed cases due to low surveillance and limited testing capacities (Chitungo *et al.*, 2020). Furthermore, the clinical COVID-19 diagnosis is even more challenging due to other endemic febrile diseases such as malaria or typhoid fever, which also act as competing priorities for frontline HCWs (Maze *et al.*, 2018). These competing priorities make it difficult for hospitals to implement triage and infectious disease treatment centres specifically dedicated for the COVID-19 pandemic (Ayebare *et al.*, 2020). This aspect is reflected by the fact that only one included study reported having a COVID-19 ward and none to have a triage system in place.

Additionally, even where cases are accurately diagnosed, self-protection of HCWs is difficult due to IPC measure limitations in infrastructure and equipment, such as overcrowded settings (van de Ruit *et al.*, 2020), in which proper distancing is more challenging and lacking supply or access to PPE material (Desai *et al.*, 2019). This lack of PPE availability or the need for increased supply was confirmed by five of the included studies. However, in contrast to studies in the UK or USA (Nguyen *et al.*, 2020), no significant association between IPC and seropositivity among HCWs in Africa was shown in our review (Abdelmoniem *et al.*, 2021; Kassem *et al.*, 2020; Mukwege *et al.*, 2021). This lack of association could arise from the assessment procedure through questionnaire only. Observation should be used in conjunction with questionnaires to realistically measure the impact of IPC on infection mitigation (Sax *et al.*, 2009).

The highest HCW prevalence rate was found in Nigeria (45.1%) and supports reports that this country has Africa’s second largest population incidence (WHO, 2021b) This high rate was also reflected in the comparative general population data that showed Nigeria to be second highest after South Africa. Providing care with minimal precautionary measures was a reported factor that is likely influencing high seroprevalence rates among HCWs in particular (Olayanju *et al.*, 2020).

Three of the included studies reporting a HCW seroprevalence <5% were conducted in April 2020, before the first wave peaked in Africa (WHO, 2021b).

Detection and subsequent isolation of SARS-CoV-2 cases is known to be especially challenging when asymptomatic rates are high. In this scoping review, asymptomatic rates were predominately >60% (Goldblatt *et al.*, 2021; Kassem *et al.*, 2020; Mostafa *et al.*, 2021; 2020; Mukwege *et al.*, 2021) and hence much higher than in a current meta-analysis (He *et al.*, 2020). These high asymptomatic rates warrant extra vigilance in case detection strategies since asymptomatic HCWs can not only infect their peers, colleagues and families but also the vulnerable group of patients they are caring for. These high asymptomatic rates are in particular worrisome in the African region where continuous mass screening of HCWs is rarely done, asymptomatic infections are broadly undetected and hence HCWs are more likely to transmit the infections onwards. Consecutively, half of the included studies recommended the implementation of routine screening of HCWs to avoid hidden onward transmission. Routine testing in practice can hence mitigate the possibility of HCWs being a potential source of infection to the vulnerable patients and protect the scarce workforce of HCWs in low-resource settings.

In the African region, significant association was shown between seropositivity and working in operating rooms, obstetrics, outpatient clinic, gynaecology and emergency department (Mostafa *et al.*, 2020; Olayanju *et al.*, 2020; Rusakaniko *et al.*, 2021). These risk areas are in accordance with results from Denmark that found emergency units to be a high-risk area (Iversen *et al.*, 2020). One included study found that nurses and non-clinical HCWs were at higher risk of seropositivity than doctors (Mostafa *et al.*, 2020), supporting our found association between seropositivity and lower level of education or the non-prioritization for IPC trainings. These associations show that non-clinical HCWs should also be included in training regardless of their level of patient contact. Interestingly, a study from Cairo found that exposure to a confirmed case at home had a significantly higher HR than exposure at work (Mostafa *et al.*, 2021), suggesting that HCWs’ non-occupational risk should also be considered. This finding supports the determined general population seroprevalence range being similar to that of HCWs. Seroprevalence was associated with higher age in three included studies (Rusakaniko *et al.*, 2021; Mostafa *et al.*, 2020; 2021), which is concerning since higher age is known to be associated with higher risk for severe disease (ECDC, 2021).

### Limitations

There are several limitations in the present scoping review. No grey literature of news and press releases were included. We searched in databases and public preprint servers to assess as much available scientific information as possible. As studies from only 11 countries could be identified, extrapolation of findings to all HCWs in the African continent could not be made. However, all African regions were represented, although data on risk factors mainly stemmed from Egyptian studies. Most studies used total population or random sampling, and none of the studies were rated to be of high risk of bias. No study explicitly included HCWs on sick leave or in quarantine leading to a potential underestimation. A valid and direct comparison between HCW and general population seroprevalence rates was challenging as neither methods nor timing of most studies were aligned.

Serological studies per se have some limitations. Given antibody kinetics, infections can be undetectable for up to 2 weeks from the onset of infection or after 3 months (Isho *et al.*, 2020). In addition to antibody kinetics, infection through immunoglobulin G testing may also be undetected as some individuals may not develop antibodies (Mostafa *et al.*, 2021). Overestimation, on the other hand, could be possible due to cross-reactive antibodies that have been detected in sub-Saharan Africa (Tso *et al.*, 2020). Test characteristics and test validity also affect seropositivity. Ten of the 16 studies used tests that met the commonly set minimum performance criteria of sensitivity ≥90% and test specificity >95% (Biocentury, 2020). These varying tests are leading to potentially divergent results that stress the need for validation in study populations, especially in African countries, where the concern of potential lower specificity of SARS-CoV-2 commercial tests has been raised (Nkuba Ndaye *et al.*, 2021). Confirmation through neutralizing antibody testing and interpretation with caution has therefore been recommended (Nkuba Ndaye *et al.*, 2021).

### Public health contribution

To our knowledge, this is the first scoping review including all preprint and published studies estimating seroprevalence and risk factors for SARS-CoV-2 seropositivity among HCWs from 11 African countries with evidence appraisal. The present scoping review has shown that data on seroprevalence in HCWs across the African continent are scarce and hence data on risk factors even scarcer. Even though there is no direct and valid comparison, this review offers the first hint that differences between seroprevalences of HCWs and the general population are not as prominent as in other regions (Nguyen *et al.*, 2020). Little standardization of study protocols, including harmonization of tests, is done, and consecutively, seroprevalence rates and risk factors are only marginally comparable between studies and countries. This scoping review therefor stands as a roadmap to compiling and understanding available data on the COVID-19 burden in HCWs across Africa, a region in need for increased investment in research (Guleid *et al.*, 2021). This roadmap provides the most up-to-date synthesis of all available research and its gaps. It is a beneficial resource for larger projects such as the planned representative national studies by the Africa CDC (Maeda and Nkengasong, 2021) and surveillance unity studies by the World Health Organization (WHO, 2020a). This serological data will help to assess the true burden and risk factors among HCWs across the African continent and hence provide knowledge to more strategically apply limited resources in response aspects such as IPC measures and vaccine programmes. Furthermore, serological data provides the possibility to make vaccination prioritization more efficient with adaptations such as prioritization of HCWs without detectible antibodies or single-dose vaccination of those who are seropositive (Krammer *et al.*, 2021). Such a strategic development of the vaccination programme could better utilize limited vaccination resources given the high seroprevalence rate of HCWs found in some African countries.

## Conclusion

This scoping review found a varying, but often high SARS-CoV-2 seroprevalence among HCWs in the African region. HCWs of older age and lower education, as well as nurses and those working in gynaecology, emergency department, outpatient clinic and operating rooms are at even higher risk. These results point to a clear need to target specific risks of HCWs, whether they arise due to their social position or due to increased exposure of certain functions/settings within the clinic. Further research is needed to better understand which factors lead to increased risk of certain cadres of health workers. Asymptomatic rates were high and half of the studies recommended routine testing of HCWs. Public health strategies should take these risk aspects and the partly high seroprevalence among HCWs into account when prioritizing IPC measures and high-risk groups for vaccine deployment.

## Supplementary Material

czab133_Supp

## Data Availability

The data underlying this article are available in the article and in its online supplementary material.

## Abbreviations

CI: Confidence interval

HCW: Healthcare worker

HR: Hazard ratio

IPC: Infection prevention and control

JBI: Joanna Briggs Institute

NA: Not applicable

NR: Not reported

OR: Odds ratio

PPE: Personal protective equipment

PRISMA-ScR: Preferred Reporting Items for systematic reviews and Meta-Analyses extension for Scoping Reviews

SARS-CoV-2: severe acute respiratory syndrome coronavirus type 2
