# Supplementary material for: Seroprevalence and risk factors of COVID-19 in healthcare workers from 11 African countries: a scoping review and appraisal of existing evidence
Source: Health Policy Plan. 2021 Nov 2;37(4):505–13. doi: 10.1093/heapol/czab133 (PMC8689910; doi:10.1093/heapol/czab133)
Supplement: czab133_Supp [file czab133_supp.zip › suppl_2.docx]

Search strategy

## SET 1: COVID

The RKI developed a SARS-CoV-2 -database in endnote X7 using the following search strings:

- 1. PubMed:
     1. Seachstring 1:

("Severe Acute Respiratory Syndrome Coronavirus 2" [Supplementary Concept] OR "COVID-19" [Supplementary Concept] OR "covid 19 diagnostic testing" [Supplementary Concept] OR "covid 19 drug treatment" [Supplementary Concept] OR "covid 19 serotherapy"[Supplementary Concept] OR "covid 19 vaccine" [Supplementary Concept] OR "Severe Acute Respiratory Syndrome Coronavirus 2"[tiab] OR ncov*[tiab] OR covid*[tiab] OR sars-cov-2[tiab] OR "sars cov 2"[tiab] OR "SARS Coronavirus 2"[tiab] OR "Severe Acute Respiratory Syndrome CoV 2"[tiab] OR "Wuhan coronavirus"[tiab] OR "Wuhan seafood market pneumonia virus"[tiab] OR "SARS2"[tiab] OR "2019-nCoV"[tiab] OR "hcov-19"[tiab] OR „novel 2019 coronavirus“[tiab] OR "2019 novel coronavirus*"[tiab] OR „novel coronavirus 2019*“[tiab] OR "2019 novel human coronavirus*"[tiab] OR „human coronavirus 2019“[tiab] OR "coronavirus disease-19"[tiab] OR "corona virus disease-19"[tiab] OR "coronavirus disease 2019"[tiab] OR "corona virus disease 2019"[tiab] OR "2019 coronavirus disease"[tiab] OR "2019 corona virus disease"[tiab] OR „novel coronavirus disease 2019“[tiab] OR „novel coronavirus infection 2019“[tiab] OR "new coronavirus*"[tiab] OR "coronavirus outbreak"[tiab] OR "coronavirus epidemic"[tiab] OR "coronavirus pandemic"[tiab] OR "pandemic of coronavirus"[tiab]) AND ("2019/12/01"[PDAT] : "2099/12/31"[PDAT])

- - 1. Seachstring 2:

("wuhan"[tiab] or china[tiab] or hubei[tiab]) AND ("Severe Acute Respiratory Syndrome Coronavirus 2"[Supplementary Concept] OR "COVID-19" [Supplementary Concept] OR "covid 19 diagnostic testing"[Supplementary Concept] OR "covid 19 drug treatment"[Supplementary Concept] OR "covid 19 serotherapy"[Supplementary Concept] OR "covid 19 vaccine"[Supplementary Concept] OR "coronavirus*"[tiab] OR "corona virus*"[tiab] OR ncov[tiab] OR covid*[tiab] OR sars*[tiab])

- 1. Embase
     1. Seachstring 1:

('severe acute respiratory syndrome coronavirus 2':ti,ab OR 'severe acute respiratory syndrome coronavirus 2'/exp OR 'covid 19'/exp OR ncov*:ti,ab OR covid*:ti,ab OR 'sars cov 2':ti,ab OR 'sars-cov-2':ti,ab OR 'sars coronavirus 2':ti,ab OR 'sars coronavirus 2'/exp OR 'severe acute respiratory syndrome cov 2':ti,ab OR 'wuhan coronavirus':ti,ab OR 'wuhan seafood market pneumonia virus':ti,ab OR sars2:ti,ab OR '2019-ncov':ti,ab OR 'hcov-19':ti,ab OR 'novel 2019 coronavirus':ti,ab OR '2019 novel coronavirus*':ti,ab OR 'novel coronavirus 2019'/exp OR '2019 novel human coronavirus*':ti,ab OR 'human coronavirus 2019':ti,ab OR 'coronavirus disease-19':ti,ab OR 'corona virus disease-19':ti,ab OR 'coronavirus disease 2019':ti,ab OR 'coronavirus disease 2019'/exp OR 'corona virus disease 2019':ti,ab OR '2019 coronavirus disease':ti,ab OR 'novel coronavirus 2019*':ti,ab OR 'novel coronavirus disease 2019':ti,ab OR 'novel coronavirus infection 2019':ti,ab OR '2019 corona virus disease':ti,ab OR 'new coronavirus*':ti,ab OR 'coronavirus outbreak':ti,ab OR 'coronavirus epidemic':ti,ab OR 'coronavirus pandemic':ti,ab OR 'pandemic of coronavirus':ti,ab OR 'severe acute respiratory syndrome coronavirus 2 vaccine'/exp OR 'covid 19 vaccine'/exp) **AND (2020:py OR 2021:py)**

- - 1. Seachstring 2:

(wuhan:ti,ab OR china:ti,ab OR hubei:ti,ab) AND ('severe acute respiratory syndrome coronavirus 2':ti,ab OR 'severe acute respiratory syndrome coronavirus 2'/exp OR 'severe acute respiratory syndrome coronavirus 2' OR 'covid*':ti,ab OR 'covid 19'/exp OR 'covid 19' OR coronavirus*:ti,ab OR 'corona virus*':ti,ab OR ncov:ti,ab OR covid*:ti,ab OR sars*:ti,ab OR 'sars coronavirus 2'/exp)

- 1. additional manual research
     1. ArRvix

title=COVID-19 OR abstract=SARS-CoV-2 OR abstract=COVID-19 OR title=SARS-CoV-2 OR title=coronavirus OR abstract=coronavirus

- - 1. BioRvix

COVID-19 OR SARS-CoV-2

- - 1. ChemRvix

Covid OR sars-cov-2

- - 1. MedRvix

COVID-19 OR SARS-CoV-2

- - 1. Preprints.org

COVID-19 and SARS-CoV-2

- - 1. ResearchSquare

https://www.researchsquare.com/coronavirus

- - 1. SSRN

<https://papers.ssrn.com/sol3/Jeljour_results.cfm?form_name=journalBrowse&journal_id=3526423&network=no>

## SET 2: Seroprevalence

The RKI endnote database was used to search for seroprevalence data using the following search terms in “contains” mode:

1. Sero:

seroepidemiologic [any field] OR sero-epidemiologic [any field] OR serologic [any field] OR seroprevalen [any field] OR sero-prevalen [any field] OR serosurve [any field] OR sero-surve [any field] OR serostud [any field] OR sero-stud [any field] OR seroposit [any field] OR sero-posit [any field] OR

1. Antibody:

antibod [any field] OR anti-bod [any field] OR "Ig G" [any field] OR "Ig M" [any field] OR immunoglobulin [any field] OR immune globulin [any field]

1. Tests:

immunoassay [any field] OR ELISA [any field] OR enzyme linked immunosorbent assay [any field] OR LFIA [any field] OR lateral flow immunoassay [any field] OR CLIA [any field] OR chemiluminescent immunoassay [any field]

Set 3: HWC:
adapted from (1) seroprevalence data on HCW was searched for.

- 1)
  - health personnel [all fields] OR healthcare personnel [all fields] OR health care personnel [all fields] OR
  - health worker [all fields] OR healthcare worker [all fields] OR health care worker [all fields] OR
  - healthcare provider [all fields] OR healthcare provider [all fields] OR health care provider [all fields] OR
- 2)
  - health practitioner [all fields] OR healthcare practitioner [all fields] OR health care practitioner [all fields] OR
  - health employee [all fields] OR healthcare employee [all fields] OR health care employee [all fields] OR
  - health professional [all fields] OR health care professional [all fields] OR healthcare professional [all fields] OR
- 3)
  - medical staff [all fields] OR medical care personnel [all fields] OR medical worker [all fields]
  - hospital staff [all fields] OR hospital personnel [all fields] OR hospital worker [all fields] OR
  - clinical staff [all fields] OR clinic staff [all fields]
- 4)
  - Doctor [all fields] OR physician [all fields] OR paramedic [all fields] OR nursing staff [all fields] OR nurse [all fields] OR nursing auxiliary [all fields] OR]
- 5)
  - Health-care personnel [all fields] OR health-care worker [all fields] OR health-care provider [all fields] OR health-care practitioner [all fields] OR health-care employee [all fields] OR health-care professional [all fields] OR health sector [all fields]

## SET 4: Africa

adapted from (2) seroprevalence data on HCWs in the African region was searched for

- Africa-Chad

Africa [any field] OR Algeria [any field] OR Angola [any field] OR Benin [any field] OR Botswana [any field] OR Burkina Faso [any field] OR Burundi [any field] OR Cameroon [any field] OR Cape Verde [any field] OR Chad [any field] OR

- Comoros-Guinea

Comoros [any field] OR Congo [any field] OR Djibouti [any field] OR Egypt [any field] OR Eritrea [any field] OR Ethiopia [any field] OR Gabon [any field] OR Gambia [any field] OR Ghana [any field] OR Guinea [any field] OR

- CIV-Mali

Ivory Coast [any field] OR Cote d’Ivoire [any field] OR Jamahiriya [any field] OR Kenya [any field] OR Lesotho [any field] OR Liberia [any field] OR Libya [any field] OR Madagascar [any field] OR Malawi [any field] OR Mali [any field] OR

- Mauritania- Reunion

Mauritania [any field] OR Mauritius [any field] OR Mayotte [any field] OR Morocco [any field] OR Mozambique [any field] OR Namibia [any field] OR Niger [any field] OR Nigeria [any field] OR Principe [any field] OR Reunion [any field] OR

- Rwanda- Tanzania

Rwanda [any field] OR Sao Tome [any field] OR Senegal [any field] OR Seychelles [any field] OR Sierra Leone [any field] OR Somalia [any field] OR St Helena [any field] OR Sudan [any field] OR Swaziland [any field] OR Tanzania [any field] OR

- Togo- Zimbabwe

Togo [any field] OR Tunisia [any field] OR Uganda [any field] OR Western Sahara [any field] OR Zaire [any field] OR Zambia [any field] OR Zimbabwe [any field]

References:

1. Ruotsalainen JH, Verbeek JH, Mariné A, Serra C. Preventing occupational stress in healthcare workers. The Cochrane database of systematic reviews. 2014(11):Cd002892.

2. Barth DD, Mayosi BM, Jabar A, Engel ME. Prevalence of group A streptococcal disease in North and Sub-Saharan Africa: a systematic review protocol. BMJ Open. 2015;5(8):e008646.
