# Supplementary material for: Seroprevalence and risk factors of COVID-19 in healthcare workers from 11 African countries: a scoping review and appraisal of existing evidence
Source: Health Policy Plan. 2021 Nov 2;37(4):505–13. doi: 10.1093/heapol/czab133 (PMC8689910; doi:10.1093/heapol/czab133)
Supplement: czab133_Supp [file czab133_supp.zip › Suppl_3_clean.docx]

| **Suppl. Table 3. Quality assessment using Joanna Briggs Institute critical appraisal tools for prevalence studies** | | | | | | | | | | |
| --- | --- | --- | --- | --- | --- | --- | --- | --- | --- | --- |
| Reference | 1.  sample frame appr. for target pop. | 2. participants sampled appr. | 3.  sample size adequate | 4.  study subjects/setting described in detail | 5.  data analysis with suff. coverage | 6.  valid methods used | 7.  standard reliable measurement | 8.  appr. statistical analysis | 9. response rate appr. | Overall |
| Abdelmoniem et al. (Abdelmoniem et al., 2021) | 1 | 1 | 1 | 1 | 1 | 1 | 1 | 0 | 0 | 7 |
| Chibwana et al. (Chibwana et al., 2020) | 1 | 0 | 1 | 1 | 1 | 1 | 1 | 1 | 0 | 7 |
| Etyang et al. (Etyang et al., 2021) | 1 | 0 | 1 | 1 | 1 | 1 | 1 | 1 | 0 | 7 |
| Fwoloshi et al. (Fwoloshi et al., 2021) | 1 | 0 | 1 | 1 | 1 | 1 | 1 | 1 | 1 | 8 |
| Goldblatt et al. (Goldblatt et al., 2021) | 1 | 0 | 1 | 1 | 1 | 1 | 1 | 0 | 0 | 6 |
| Halatoko et al. (Halatoko et al., 2020) | 1 | 1 | 1 | 1 | 1 | 0 | 1 | 1 | 0 | 7 |
| Kammon et al. (Kammon et al., 2020) | 1 | 1 | 1 | 1 | 1 | 0 | 1 | 0 | 0 | 6 |
| Kassem et al. (Kassem et al., 2020) | 1 | 1 | 1 | 1 | 1 | 1 | 1 | 0 | 0 | 7 |
| Majiya et al. (Majiya et al., 2020) | 1 | 1 | 0 | 0 | 1 | 1 | 1 | 0 | 0 | 5 |
| Mostafa et al.  2020 (Mostafa et al., 2020) | 1 | 1 | 1 | 1 | 1 | 0 | 1 | 1 | 1 | 8 |
| Mostafa et al.  2021 (Mostafa et al., 2021) | 1 | 1 | 1 | 1 | 1 | 0 | 1 | 1 | 0 | 7 |
| Mukhtar et al. (Mukhtar et al., 2021) | 1 | 0 | 1 | 1 | 1 | 1 | 1 | 1 | 1 | 8 |
| Mukwege et al. (Mukwege et al., 2021) | 1 | 1 | 1 | 1 | 1 | 0 | 1 | 1 | 1 | 8 |
| Olayanju et al. (Olayanju et al., 2020) | 1 | 1 | 1 | 1 | 1 | 0 | 1 | 1 | 0 | 7 |
| Rusakaniko et al. (Rusakaniko et al., 2021) | 1 | 1 | 1 | 1 | 1 | 0 | 1 | 1 | 1 | 8 |
| Salem et al. (Salem et al., 2021) | 1 | 1 | 1 | 1 | 0 | 1 | 1 | 0 | 0 | 6 |
| 0 indicates „no“, 1 indicates „yes“. A higher score means higher overall quality. | | | | | | | | | | |
