# Supplementary material for: Seroprevalence and risk factors of COVID-19 in healthcare workers from 11 African countries: a scoping review and appraisal of existing evidence
Source: Health Policy Plan. 2021 Nov 2;37(4):505–13. doi: 10.1093/heapol/czab133 (PMC8689910; doi:10.1093/heapol/czab133)
Supplement: czab133_Supp [file czab133_supp.zip › Suppl_4..docx]

Meta-analysis

Statistical analysis

The total number of HCWs and those seropositive were extracted. Seroprevalence and 95% confidence interval was calculated. Before pooling, seroprevalence was transformed applying the Freeman-Tukey double arcsine method (Barendregt et al., 2013). Heterogeneity was assessed using inconsistency statistic (I^2^), with >75% indicating high heterogeneity (Higgins et al., 2003). A random effect model was applied to estimate pooled seroprevalence as heterogeneity was very high. Studies were explored in meta-regression and subgroup analysis. Two subgroups were created according to diagnostic test standards (sensitivity levels of >/< 90% and specificity levels of >/< 95%).

Results

One study was excluded from meta-analysis, as it was a follow-up cohort study only reporting seroconversion rate (Mostafa et al., 2021). Heterogeneity between studies was very high (I^2^=99%, p<0.01). The estimated overall seroprevalence was 12.6% (95%CI 6.3-20.6)(Figure 1). Subgroup analysis showed no significant difference between studies which met testing standards (test sensitivity >90% and test specificity >95% )(11.8, 95%CI 6.0-19.3) compared to studies that did not (13.5, 95%CI 1.7-33.5, p=0.86).


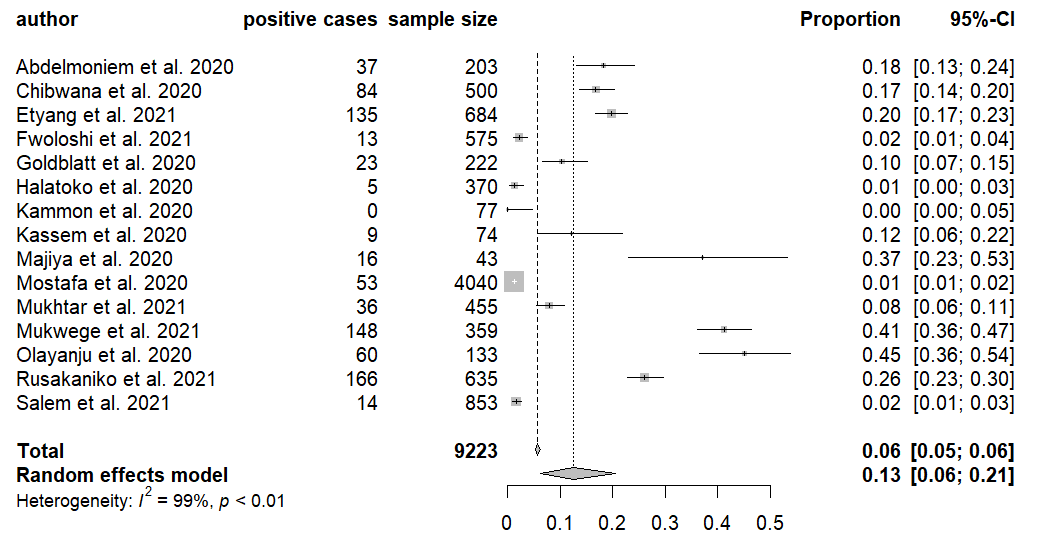


Figure 1. Forest plot of seroprevalence with 95%CI.

BARENDREGT, J. J., DOI, S. A., LEE, Y. Y., NORMAN, R. E. & VOS, T. 2013. Meta-analysis of prevalence. *J Epidemiol Community Health,* 67**,** 974-8.

HIGGINS, J. P., THOMPSON, S. G., DEEKS, J. J. & ALTMAN, D. G. 2003. Measuring inconsistency in meta-analyses. *Bmj,* 327**,** 557-60.

MOSTAFA, A., KANDIL, S., EL-SAYED, M. H., GIRGIS, S., HAFEZ, H., YOSEF, M., SABER, S., EZZELARAB, H., RAMADAN, M., ALGOHARY, E., FAHMY, G., AFIFI, I., HASSAN, F., ELSAYED, S., REDA, A., FATTUH, D., MAHMOUD, A., MANSOUR, A., SABRY, M., HABEB, P., EBEID, F. S. E., ELANWAR, A., SALEH, A., MANSOUR, O., OMAR, A. & EL-METEINI, M. 2021. SARS-CoV-2 seroconversion among 4040 Egyptian healthcare workers in 12 resource-limited healthcare facilities: A prospective cohort study. *International Journal of Infectious Diseases*.
