# Supplementary material for: Seroprevalence and risk factors of COVID-19 in healthcare workers from 11 African countries: a scoping review and appraisal of existing evidence
Source: Health Policy Plan. 2021 Nov 2;37(4):505–13. doi: 10.1093/heapol/czab133 (PMC8689910; doi:10.1093/heapol/czab133)
Supplement: czab133_Supp [file czab133_supp.zip › Suppl_5.docx]

| **Suppl. Table 5. Main characteristics of studies and antibody tests** | | | | | | | | | | | | | | |
| --- | --- | --- | --- | --- | --- | --- | --- | --- | --- | --- | --- | --- | --- | --- |
| Reference | Publication | City country | Setting | Data collection mm/yy | Sample  size (N) | Study  design | Sampling  method | males  (%) | Age  (years) | Antibody test kit | Type of antibody | Type of test | Sensitivity (%) | Specificity (%) |
| Abdelmoniem et al. (Abdelmoniem et al., 2021) | journal | Cairo Egypt | hospital,  emergency dep. | 06/20 | 203 | prevalence | total population | 51.2 | mean 31.9  (SD 6.6) | Artron Laboratories | IgM/IgG | rapid | 93.4 | 97.7 |
| Chibwana et al. (Chibwana et al., 2020) | preprint | Blantyre Malawi | hospital | 05/20 - 06/20 | 500 | prevalence | convenience | 47.0 | median 31  (IQR 20-64) | Omega diagnostics Mologic | IgG | ELISA | 94-96.0 | 97.0 |
| Etyang et al. (Etyang et al., 2021) | preprint | Kilifi, Busia, Nairobi, Kenya | hospital | 07/20-12/20 | 684 | prevalence | convenience | 46.0 | mean 35 (SD 11) | Krammer Enzyme | IgG | ELISA | 92.7 | 99.0 |
| Fwoloshi et al. (Fwoloshi et al., 2021) | journal | 6 districts Zambia | health facilities | 07/20 | 575 | prevalence | convenience | 34.0 | median 31.5 (IQR 26-40) | PerkinElmer, Waltham, Massachusetts | IgG | ELISA | 90 | 100 |
| Goldblatt et al. (Goldblatt et al., 2021) | journal | Cape Town South Africa | hospital,  pediatric dep. | 06/20 - 08/20 | 222 | prevalence | convenience | 21.2 | median 41  (IQR 19-67) | Epitope Diagnostics (EDI), USA | IgG | ELISA | 100.0 | 88.7 |
|  |  |  |  |  |  |  |  |  |  | Mulitplexed assay (Meseo Scale Discovery) | IgG | CLIA | 97.4 | 96.2 |
| Halatoko et al. (Halatoko et al., 2020) | journal | Lomé Togo | NR | 04/20 - 05/20 | 370 | prevalence | random | 48.9 | < 36  42.7% | BIOLIM Hangzhou Clungene Biotech | IgM/IgG | rapid | 72.9 | 85.0 |
| Kammon et al. (Kammon et al., 2020) | preprint | Alzintan Libya | hospital | 04/20 - 05/20 | 77 | prevalence | random | 70.1 | < 40  76.6% | One Step Novel Coronavirus Guangzhou Wondfo | IgM/IgG | rapid | 86.4 | 99.6 |
| Kassem et al. (Kassem et al., 2020) | journal | Cairo Egypt | hospital,  gastroentero. dep. | 06/20 | 74 | prevalence | total population | 40.5 | median 32 (IQR 23-48) | Artron Laboratories | IgM/IgG | rapid | 93.4 | 97.7 |
| Majiya et al. (Majiya et al., 2020) | preprint | Niger State Nigeria | NR | 06/20 | 43 | prevalence | random | 51.2 | NR | COVID-19 IgG and IgM Rapid Test Kit | IgM/IgG | rapid | 98.8 | 98.0 |
| Mostafa et al.  2020 (Mostafa et al., 2020) | journal | Cairo Egypt | hospital | 04/20 - 05/20 | 4040 | prevalence | total population | 38.5 | < 40  72.8% | Artron Laboratories | IgM/IgG | rapid | 83.3 | 100.0 |
| Mostafa et al.  2021 (Mostafa et al., 2021) | journal | Cairo Egypt | hospital | 05/20 - 06/20 | 2282 | cohort | total population | 34.7 | median 32  (IQR 27-42) | Artron Laboratories | IgM/IgG | rapid | 83.3 | 100.0 |
| Mukhtar et al. (Mukhtar et al., 2021) | journal | Cairo Egypt | hospital | 05/20 - 06/20 | 455 | prevalence | convenience | 52.5 | mean 32.8 (SD 8.3) | Standard-Q Covid-19 SD Biosensor, Korea | IgM/IgG | rapid | 94 | 95 |
|  |  |  |  |  |  |  |  |  |  | iFlash-SARS-CoV-2, Biotech, Shenzhen | IgG | CLIA | 90 | 95 |
| Mukwege et al. (Mukwege et al., 2021) | journal | Bukavu, DRC | hospital | 07/20 – 08/20 | 359 | prevalence | total population | 50.7 | mean 43.1 (SD 11.3) | QuickZen COVID-19 Kit | IgM/IgG | rapid | 71.1 | 100.0 |
|  |  |  |  |  |  |  |  |  |  | Euroimmun assay (Luebeck, Germany) | IgG | ELISA | 61.7 | 98.6 |
| Olayanju et al. (Olayanju et al., 2020) | journal | Ibadan Nigeria | hospital | 04/20 | 133 | prevalence | random | 47.4 | < 41  76.0% | Elabscience Biotechnology (Spike-Protein) | IgG | ELISA | NR | NR |
| Rusakaniko et al. (Rusakaniko et al., 2021) | journal | Bulawayo, Zimbabwe | health facilities | 06/20 | 635 | prevalence | total population | 26.1 | median 40 (IQR 32-52) | Wuhan UNscience Biotechnology Companies | IgM/IgG | rapid | 98.5 | 88.2 |
|  |  |  |  |  |  |  |  |  |  | Standard-Q Covid-19 Duo test SD Biosensor | IgM/IgG | rapid | 76.7 | 98.8 |
| Salem et al. (Salem et al., 2021) | journal | Nouakchott, Mauritania | hospital | 05/20 | 853 | prevalence | total population | 60.3 | mean 39 (range 20-60) | Biotime (Xiamen Biotime Biotechnology Co.) | IgM/IgG | rapid | 96.4 | 98.7 |
