# Supplementary material for: Seroprevalence and risk factors of COVID-19 in healthcare workers from 11 African countries: a scoping review and appraisal of existing evidence
Source: Health Policy Plan. 2021 Nov 2;37(4):505–13. doi: 10.1093/heapol/czab133 (PMC8689910; doi:10.1093/heapol/czab133)
Supplement: czab133_Supp [file czab133_supp.zip › Response to reviewers_HCWsero_final.docx]

# Response to reviewers

26.10.2021

Reviewer: 1

Dear authors,

thanks for your revised version. It certainly is a major improvement that you dropped the meta-analysis.

**Response:** Thank you!

But, in my opinion, thé major problem with your paper remains. I fear that my first review was not explicit enough, so that you did not understand that my feed-back called for a fundamental rethink of your paper: “a valid comparison between seroprevalence among HCWs with the seroprevalence in comparable samples of the general population.” You did not do that, not contrasting studies among HCWs with data available in the general population, arguing that such data are simply not available, and sticking to a statement in a paper from very early in the epidemic, making the unsubstantiated claim that seroprevalence is 3 to 12 time higher among HCWs. Comparing with pooled worldwide prevalence certainly is not a good substitute; not at all.

This major problem is clearly reflected in the first paragraph of the discussion:

“The overall seroprevalence among HCWs across eleven Africa countries varied widely and was high in comparison with a recently reported pooled prevalence of 8.7% worldwide (95% CI: 6.7-10.9%) and 8.5% in Europe (95% CI: 5.8-11.6%) (Galanis et al., 2020). This seroprevalence is especially high in consideration of Africa’s low incidence rate and community seroprevalence rate (Rostami et al., 2020), but reflects recent data from a review combining the general population and specific working groups (Chisale et al., 2021). Seroprevalence in the general population was only available for three countries and in Kenya e.g. considerably lower than seroprevalence among HCWs (Kenya HCW: 20.8% general population 9.1%) (Arora et al., 2021, Etyang et al., 2021).”

This summary is quite flawed, as recent evidence shows that throughout sub-Saharan Africa seroprevalence in the general population in urban areas is not low at all. The fact that only three nation-wide sero-prevalence studies are available, does not invalidate the many other sub-national studies, which show ample evidence of surprisingly high seroprevalence, especially in urban areas; and rates among HCWs should be compared to those (as most studies on HCWs are also done in urban areas).

It gives the impression that you are selectively looking for data to substantiate a position, rather than trying to understand the reality from all data available.

**Response:** Thank you for explaining your concern again and being more explicit. We apologize for not having fully understood your concern earlier. We aim to assess existing evidence for seroprevalence and risk factors for healthcare workers across the African region and we agree with you that addition of general population seroprevalence rates can give essential context. Many of studies available on general population were recently released after the writing of this manuscript. Comparison of studies from different time periods would skew the results, so we only included studies from the same time period. As such we have updated and changed the manuscript accordingly to make such a comparison. A valid and direct comparison was challenging as neither methods nor timing of most studies are aligned, we have, however, done a thorough of a comparison as is possible, and added the respective sections:

- Introduction
  - P3L6ff: ‘…, as well as how these seroprevalence rates compare with the respective general population.’
  - Respective sentences on P2L4 “infection rate in HCWs is reported to be as much as twelve times higher” were taken out.
- Methods
  - P3L24ff: ‘Additionally, to put the HCW seroprevalences into context, general population seroprevalences were gathered from the SeroTracker database for all countries included on October 24^th^, 2021.’;
  - P4L12ff: ‘Selection of studies for general population seroprevalence was restricted to the respective counties and overall study period’
- Results
  - P8L22ff: ‘General population seroprevalence comparison: Seroprevalence in the general population in the included countries ranged between 0% (Elfakhri et al., 2020) to 44.6% (Marion et al., 2021). For four countries, there was no general population data available: Malawi, Mauritania, Togo and Zimbabwe. Seroprevalence in the other included countries ranged from 16.6% (Nkuba et al., 2021) to 40.8% (Philippe et al., 2021) in DRC, from 29.8% (Girgis et al., 2021) to 41.0% (Musa et al., 2021) in Egypt, from 1.3% (Lucinde et al., 2021) to 34.7% (Ngere et al., 2021) in Kenya, from 0% (Elfakhri et al., 2020) to 4.2% (Kammon et al., 2020) in Libya, from 16.1% (Okpala et al., 2021) to 42.0% (Ijeoma et al., 2021) in Nigeria, from 23% (Kleynhans et al., 2021) to 44.6% (Marion et al., 2021) in South Africa, and from 2.1% (Mulenga et al., 2021) to 8.2% (Hines et al., 2021) in Zambia.’
- Discussion
  - P10L1ff: ‘The overall seroprevalence among HCWs across eleven Africa countries varied widely reflecting recent data from a systematic review of seroprevalence of anti-SARS-CoV-2 antibodies in Africa which combined the general population and specific working groups (Chisale et al., 2021). Direct comparison with the general population is challenging and was not the primary aim of the current manuscript, but data available from the general population in the eleven included countries suggest highly varying seroprevalences similar to those of HCWs with rates between 0% to 44.6%.’;
  - P11L18ff: ‘This high rate was also reflected in the comparative general population data which showed Nigeria to be second highest after South Africa. Providing care with minimal precautionary measures was a reported factor that is likely influencing high seroprevalence rates among HCW in particular (Olayanju et al., 2020).’
  - P12L24 ‘This finding supports the determined general population seroprevalence range being similar to that of HCWs.’
  - Limitations P13L11: ‘A valid and direct comparison between HCW and general population seroprevalence rates was challenging as neither methods nor timing of most studies were aligned.’
  - Public health contribution P14L5ff ‘Even though there is no direct and valid comparison, this review offers the first hint that differences between seroprevalences of HCWs and the general population are not a prominent as in other regions’

I will not make any further comments, they might distract from this one, which I consider fundamental.

**Response:** Thank you very much. We feel that the responses to your comments above have enriched the manuscript. Your experience is highly valued and we welcome any further comments.

Reviewer: 2

The authors have addressed all of my concerns. Thank you.

**Response:** Thank you!
